# Sequential Surgical Correction of Macrodactyly: A Case Report and Literature Review

**DOI:** 10.7759/cureus.68913

**Published:** 2024-09-07

**Authors:** Aurelio Pio Russo, Monika Szilveszter, Călin Șuta, Camelia David, Vlad Vunvulea, Ylenia Pastorello, Zsuzsánna Simon-Szabó, Sándor Incze-Bartha, Lóránd Dénes, Zsuzsánna Incze-Bartha

**Affiliations:** 1 Faculty of Medicine, George Emil Palade University of Medicine, Pharmacy, Science and Technology of Târgu Mureș, Târgu Mureș, ROU; 2 Department of Plastic and Reconstructive Surgery, Emergency County Hospital Târgu Mureș, Târgu Mureș, ROU; 3 Department of Plastic and Reconstructive Surgery, Dr. Fogolyán Kristóf Emergency County Hospital, Sfântu Gheorghe, ROU; 4 Department of Anatomy and Embryology, George Emil Palade University of Medicine, Pharmacy, Science and Technology of Târgu Mureș, Târgu Mureș, ROU; 5 Department of Pathophysiology, George Emil Palade University of Medicine, Pharmacy, Science and Technology of Târgu Mureș, Târgu Mureș, ROU; 6 Department of Orthopedic Surgery and Traumatology, Dr. Fogolyán Kristóf Emergency County Hospital, Sfântu Gheorghe, ROU

**Keywords:** macrodactyly, orthopedics, overgrowth, pediatric surgery, personalized treatment, plastic surgery

## Abstract

This report discusses the case of a young female patient diagnosed with macrodactyly of the toes, a condition that significantly affected her daily life. From the age of three to 11, she underwent treatment due to the severe impact of her deformity, particularly on her ability to move comfortably and wear suitable footwear. The patient’s macrodactyly presented a complex clinical challenge, necessitating multiple surgical procedures to manage it effectively. These surgeries included soft tissue reduction to decrease the bulk of the enlarged digits, epiphysiodesis to halt the growth of the affected bones, and amputations to address the disproportionate enlargement of the toes. Each surgical intervention was aimed at improving both the function and appearance of the affected foot, with a focus on enhancing the patient’s mobility and comfort.

Despite the difficulties associated with recovery, the patient showed significant improvements in her ability to walk and in the aesthetic appearance of her foot. This case underscores the importance of developing individualized treatment plans that consider the unique needs of each patient and setting realistic expectations for outcomes. It also highlights that, while surgical interventions can lead to functional and cosmetic benefits, the extent of these improvements may be limited due to the inherent complexities of macrodactyly. The case calls attention to the need for ongoing research and the accumulation of clinical experience to refine treatment approaches for macrodactyly. Such advancements are crucial for optimizing therapeutic outcomes and improving the quality of life for patients affected by this rare condition.

## Introduction

Macrodactyly is a rare congenital disorder characterized by non-hereditary isolated overgrowth of the digits. The deformity has various names: localized gigantism, megalodactyly, macrosomia, macrodystrophia lipomatosa, macrodactylia lipomatosa, and digital gigantism [1-5]. When making the diagnosis, differentiation should be determined between idiopathic macrodactyly and secondary hypertrophy. The real pathogenesis of macrodactyly remains unknown. Several theories have been proposed over time, including dysfunction of intrauterine growth-inhibiting factors, lipomatous degeneration, peripheral nerve dysfunction, and neurofibromatosis, but none have been conclusively proven. However, in children, overgrowth of a limb or digit has been observed as part of several congenital pathologies. Such abnormalities are found in Proteus, Klippel-Trenaunay-Weber, Ollier, and Maffucci syndromes, neurofibromatosis, congenital hemi-hyperplasia, hemangiomas, fibrolipomatous hamartomas, and tumors. In these conditions, hypertrophy is limited to specific tissues [6].

In macrodactyly, all tissues of the digits - skin, subcutis, muscles, tendons, and phalanges - can overgrow. The original definition spared the metacarpus and metatarsus, but several studies described their hypertrophy as well [7-12]. The deformity can affect one or more digits, including their rays. Bilateral occurrence is rare, and simultaneous involvement of upper and lower limb digits is even rarer. Other bony deformities are often associated with macrodactyly, the most common being syndactyly, polydactyly, polydactyly, and exostosis [13].

There are two forms of macrodactyly: static and progressive. In the static form, a significant size difference between the digits is present at birth, but these proportions remain unchanged with growth. In the progressive form, there is no pronounced difference in size at birth, but the affected parts disproportionately outgrow the unaffected ones over time [1]. Macrodactyly of the toes is less common compared to the one of the hands. This deformity can severely limit children’s movement and walking in everyday life, rarely causing pain but making it difficult to wear shoes. Static and progressive forms require different treatment approaches. In static cases where the deformity is minimal, treatment may be conservative: wearing shoes tailored to the individual’s shape [14]. Progressive forms require surgical treatment. Due to the variability of the pathology, surgical interventions are complex and may be multiple due to evolution. The goal of treatment is a pain-free, shoe-wearable, normal-sized foot. The surgical techniques target both bony and soft tissue corrections: soft tissue resections, osteotomies or ostectomies, epiphysiodeses, partial or total phalanx amputations, and ray resections [14-18].

According to the available literature, the prevalence of macrodactyly of the toes is 1/18.000 [4,19,20]. Due to the rarity of this deformity, there are only a small number of patient studies or case reports in the literature. There is no clear data on the time or type of surgical treatment. A major contributing factor is the extreme variability of the clinical picture, which ranges from a minimally enlarged toe to a gigantic multi-ray non-functional foot. Depending on the clinical presentation and evolution, the timing and technique of surgical treatment of each patient should be personalized. The case and seven-year follow-up of a young female patient with macrodactyly is presented here.

## Case presentation

Our patient was treated and followed up between the ages of three and 11. The child was born full-term from a second physiological pregnancy without perinatal problems. At birth, a deformity of the left foot was observed: the hallux and second toe were normal in shape but larger in size. At the time of diagnosis, no other comorbidities were found either clinically or by genetic testing, confirming the diagnosis of true macrodactyly. The changes observed at birth displayed further growth during the first years of life, leading to the diagnosis of progressive macrodactyly. The deformity was easily tolerated; the larger size of the toes did not impede the little child's movements, and the deformity was painless.

**Figure 1:**
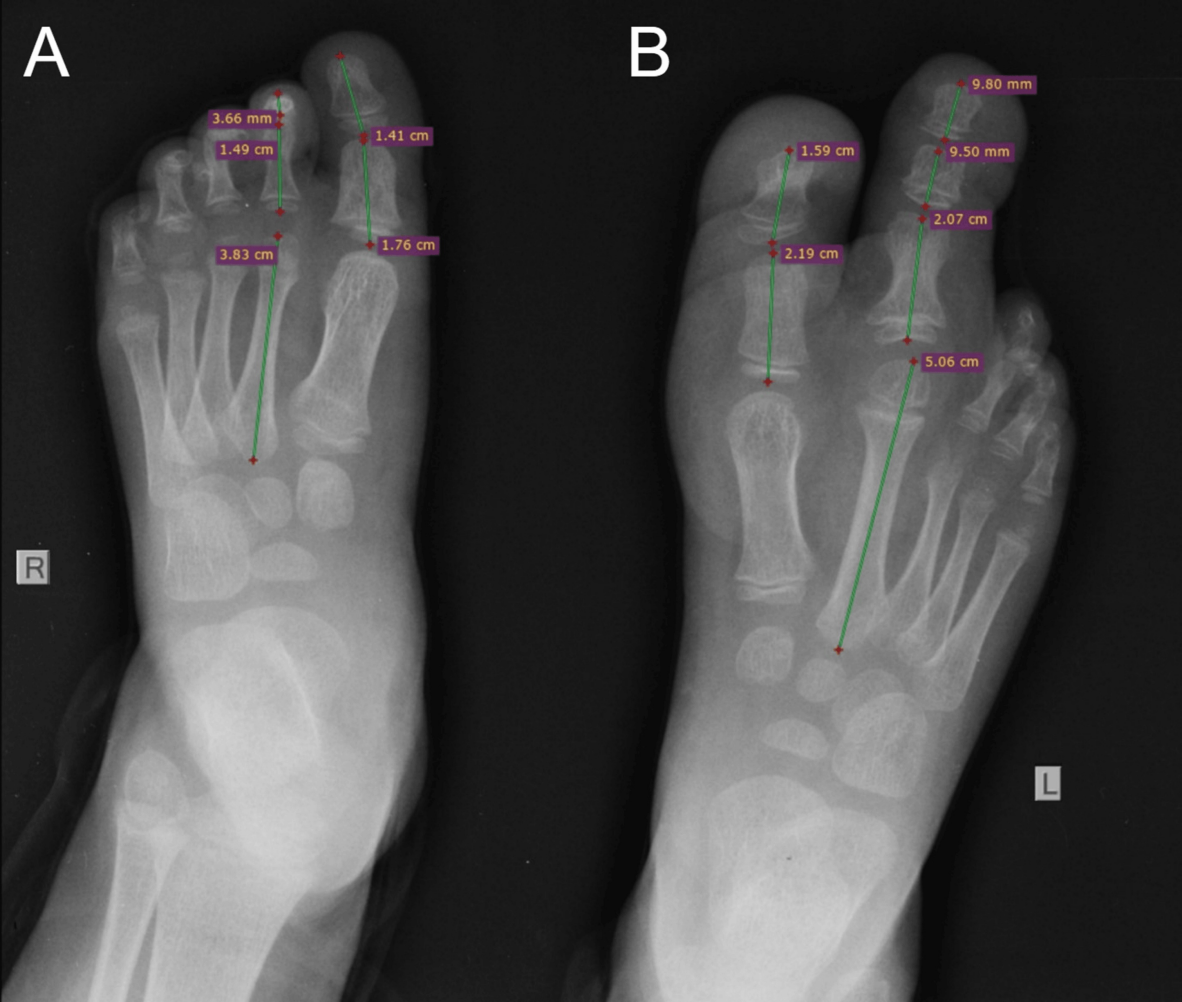
Antero-posterior (AP) X-ray at three years of age. Unaffected right foot (A) compared with the left macrodactyly one (B).

Our first clinical assessment was carried out at the age of three years. At this time, the left forefoot and the first two toes were significantly larger but normal in shape compared to the right foot. The involved toes were disproportionate in size to each other and to the other toes, with the second toe being larger than the hallux. On clinical examination, the plantar fat pad under the first and second rays of the left foot was thicker. The toenails appeared normal, except for the hallux which showed persistent and recurrent nail ingrowth after treatment. By this time, finding appropriate footwear and wearing shoes had become problematic. X-ray evidenced how the second toe outgrew the other toes by 2.7 cm and the hallux by 1 cm, which was also hypertrophic. The first metatarsus was non-hypertrophic, but an enlarged plantar fat pad was present beneath. The metatarsus and phalanges of the second ray and the phalanges of the hallux were markedly larger in length and width. The ratios between macrodactyly and normal phalanges were 1.2 for the hallux, 1.1 for the proximal phalanx and 1.3 for the distal one. For the second toe phalanges, the ratios were 2.5 and 2.8, respectively, with the second metatarsus showing an intermediate ratio of 1.32 (Figure 1).

Given the pathology, functionality, and growth rates, the selected treatment at the time was amputation of the second toe from the base of the proximal phalanx. Efforts were made to maintain a flexor-extensor traction balance so that the toe-off phase of footsteps remained robust. The complexity of the surgery was compounded by the reduction of the plantar fat pat through longitudinal plantar incision and the removal of the big toenail. There were no postoperative healing complications.

**Figure 2:**
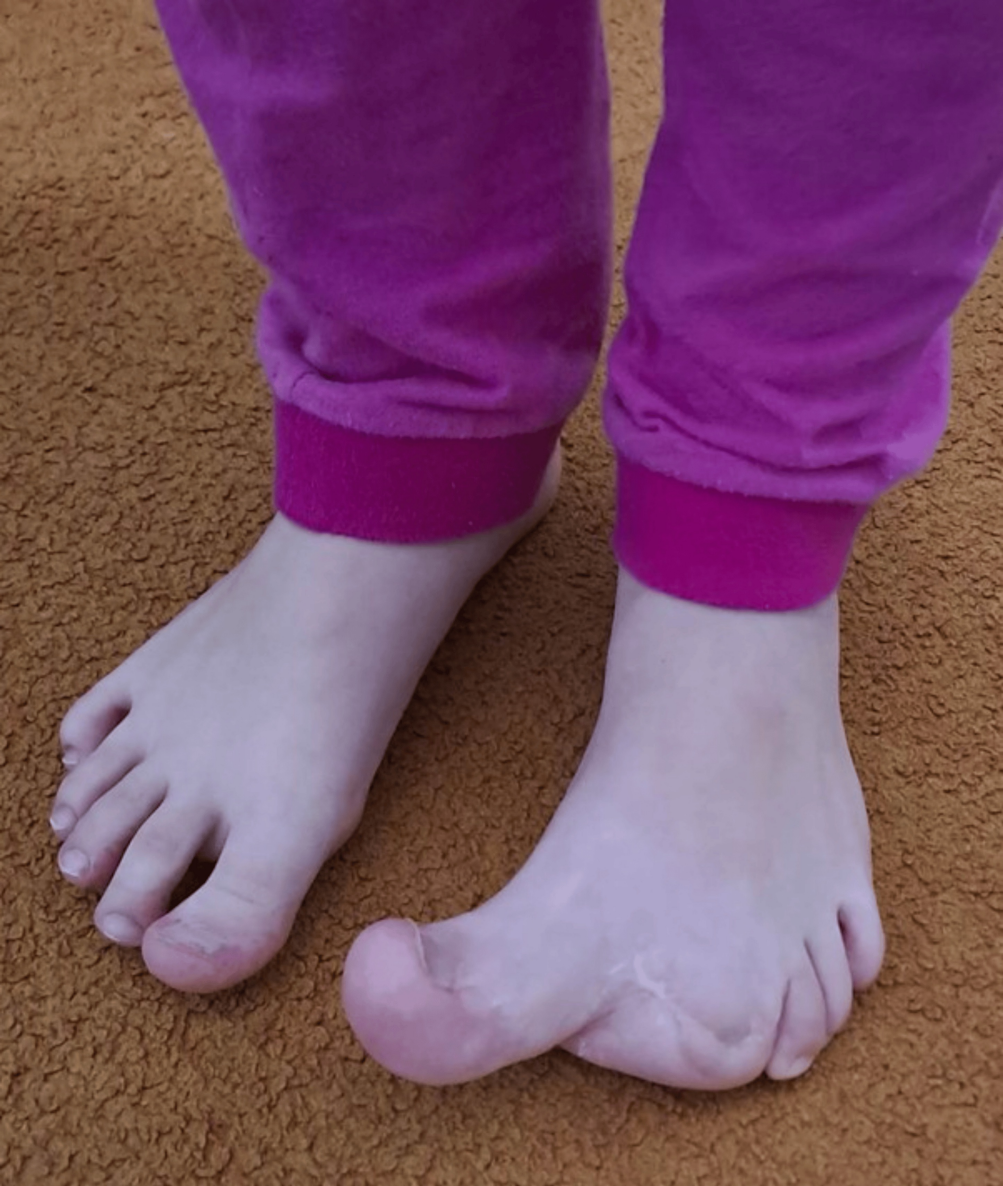
Clinical picture at the age of six.

Our long-term treatment plan included closing the growth plate of the macrodactyly bones when they reached the size of the corresponding phalanges and metatarsals of the same-sex parent. By the age of six, the bones of the patient had almost reached the size of her mother’s (Figure 2).

**Figure 3:**
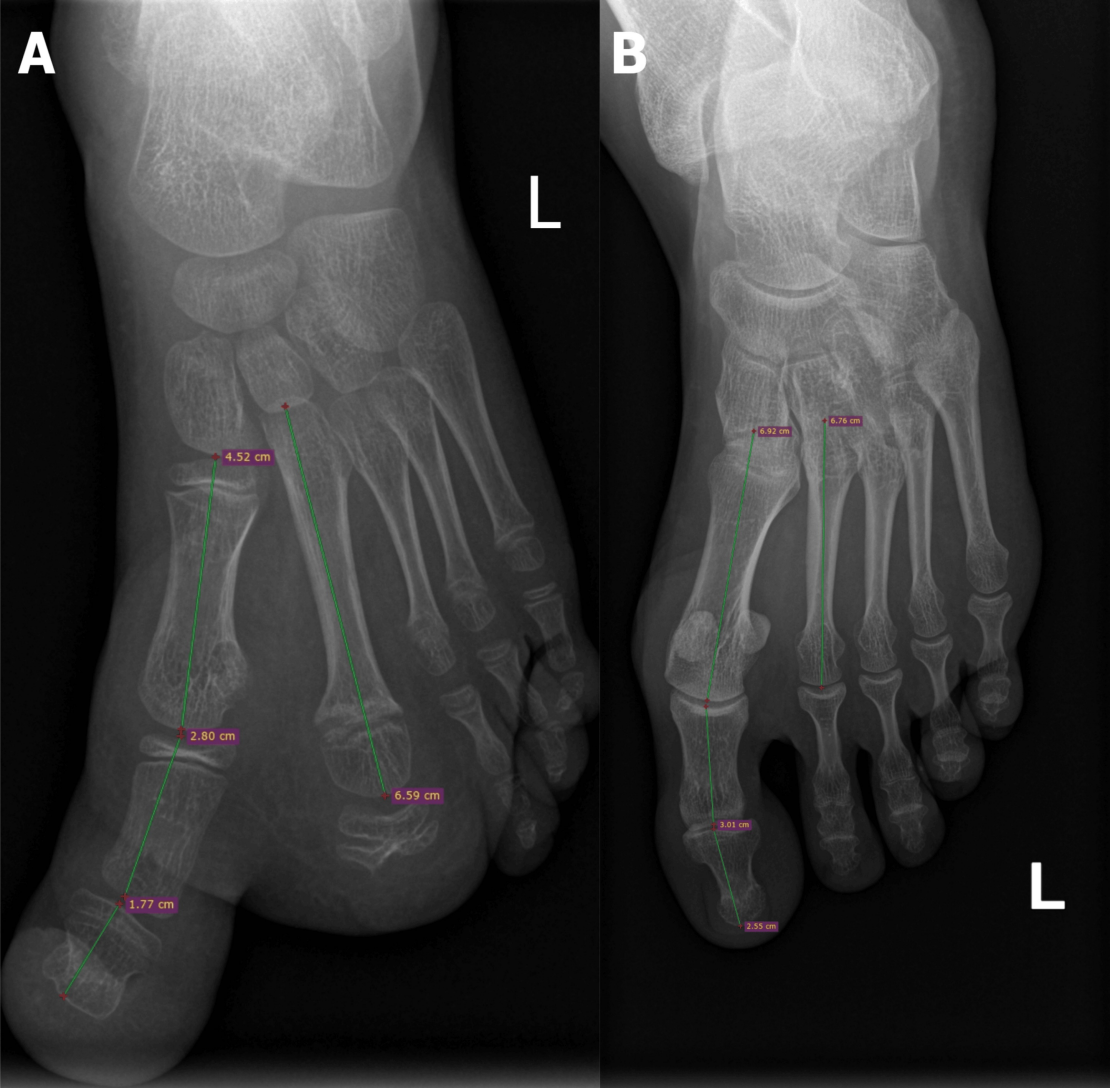
Antero-posterior (AP) X-ray of the patient's left foot at six years of age (A) compared to the left foot of the girl's mother (B). Patient's left foot first metatarsal, hallux, and second metatarsal measurements (A) compared to the mother's left foot (B).

The antero-posterior (AP) radiograph taken at that time shows nearly identical bone sizes in the feet of the patient and her mother (Figure 3).

This size congruence guided the next treatment phase, where percutaneous epiphysiodesis was performed on the proximal and second metatarsal growth cartilage of the hallux. Given the growth rate of the second metatarsus, the epiphysiodesis was stabilized with two cross-placed screws. No complications were observed in the postoperative period.

**Figure 4:**
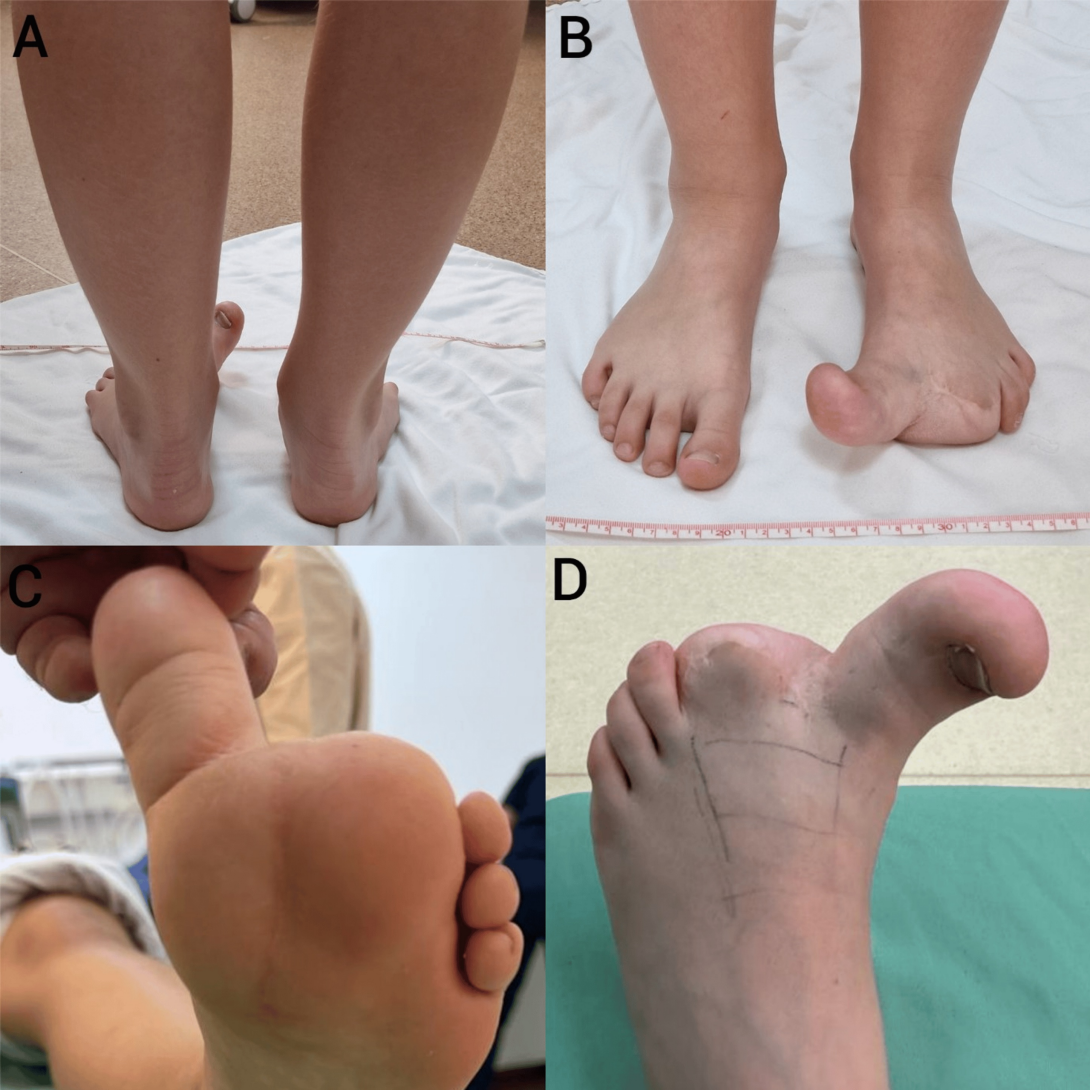
Clinical appearance of the left foot at 10 years of age. A-posterior view; B–anterior view; C–inferior view; D–superior view.

Four years after the epiphysiodesis, a new consultation was necessary due to the progression of the deformity: the length of the patient’s left hallux had exceeded the right one by three shoe sizes, accompanied by a significant enlargement of the plantar fat pad. This made wearing shoes and finding appropriate footwear challenging. During motion testing and passive motion, minimal flexion-extension was induced on the hallux joints. In all the enlarged areas, the skin was thicker and firm. Upon examination, the width of the left foot at the level of the metatarsal head was 3 cm greater than the right one, and the height of the medial plantar edge was several times greater than the lateral one. The plantar fat pad was located below the first and second rays, thus lifting the hallux from the ground and leading to support loss when walking. Also, the subcutis was thicker dorsally on the ray of the first and second toes (Figure 4).

**Figure 5:**
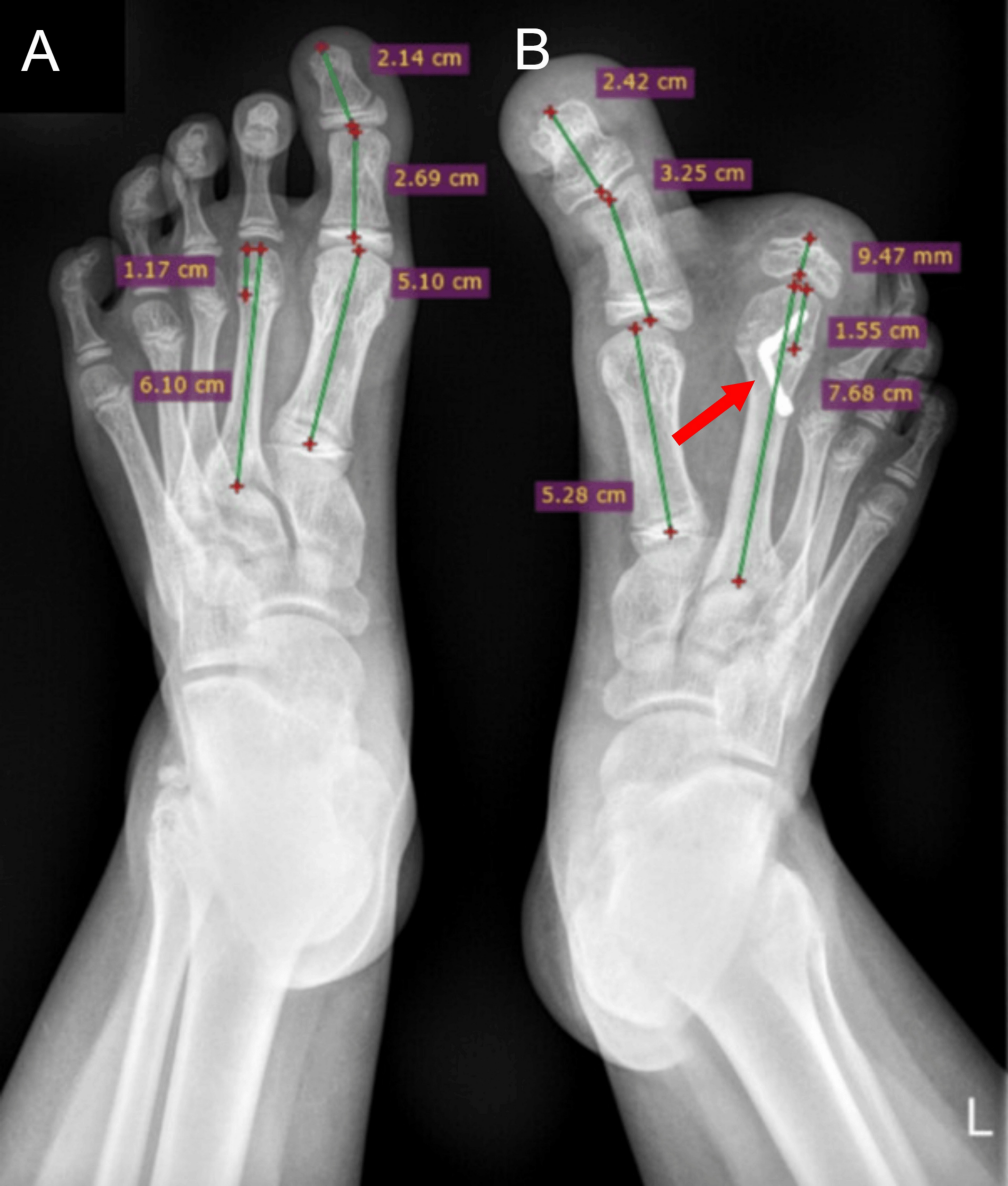
Antero-posterior (AP) X-ray of the feet at 10 years of age. Right unaffected foot of the patient (A) compared with the left macrodactyly foot (B) with visible epiphysiodesis fixing screws (red arrow).

On X-ray, the ratio between macrodactyly and normal areas was of 1.25 for the second metatarsals, followed by the proximal hallux phalanx of 1.2, and the distal hallux phalanx of 1.1 (Figure 5).

These values show a slower growth rate compared to the values measured at three years of age: the aspect ratio of the second metatarsus decreased from 1.32 to 1.25. The aspect ratios of the macrodactyly foot at three and 10 years old, together with the comparison between the patient’s left foot at six years and the mother’s left foot at the same time, are shown in Table 1. Even though the bone growth had slowed down, the presence of epiphysiodesis screws made impossible the quantification of the natural growth rate. In the distal metaphysis of the metatarsus, the screws retained their position, but the metatarsal head’s bone tissue had grown around them. The measured forefoot angles on radiographs were as follows: the first intermetatarsal angle was of 20°, while the metatarsal spread angle was of 28°.

**Table 1:** Aspect ratios of the macrodactyly foot at different years of age. The right foot is used as reference, as being unaffected. Aspect ratio comparing the left foot of the patient (six years old) and the left foot of her mother at the same time. MF: Macrodactyly Foot; RF: Right Foot

Patient				Patient and Mother- Left foot comparison			
Age	Foot area	Measurements	Aspect Ratio (Macrodactyly-to-Normal)	Age	Foot area	Measurements	Patient-mother Aspect Ratio
3	Hallux Phalanges	MF: 2.19cm proximal; 1.59cm distal	1.1 proximal; 1.3 distal	Patient 6	Hallux Phalanges	2.80cm proximal; 1.77cm distal	Hallux phalanges: 0.93 proximal; 0.69 distal - Second metatarsus: 0.97
		RF: 1.76cm proximal; 1.41cm distal					
	Second Toe Phalanges	MF: 2.07cm proximal; 9.80mm distal	2.5 proximal; 2.8 distal		Second Metatarsus	6.59cm	
		RF: 1.49cm proximal; 3.66mm distal					
	Second Metatarsus	MF: 5.06cm	1.32	Mother N/A	Hallux Phalanges	3.01cm proximal; 2.55cm distal	
		RF: 3.83cm					
10	Hallux Phalanges	MF: 3.25cm proximal; 2.42cm distal	1.2 proximal; 1.1 distal		Second Metatarsus	6.76cm	
		RF: 2.69cm proximal; 2.14cm distal					
	Second Metatarsus	MF: 7.68cm	1.25				
		RF: 6.10cm					

**Figure 6:**
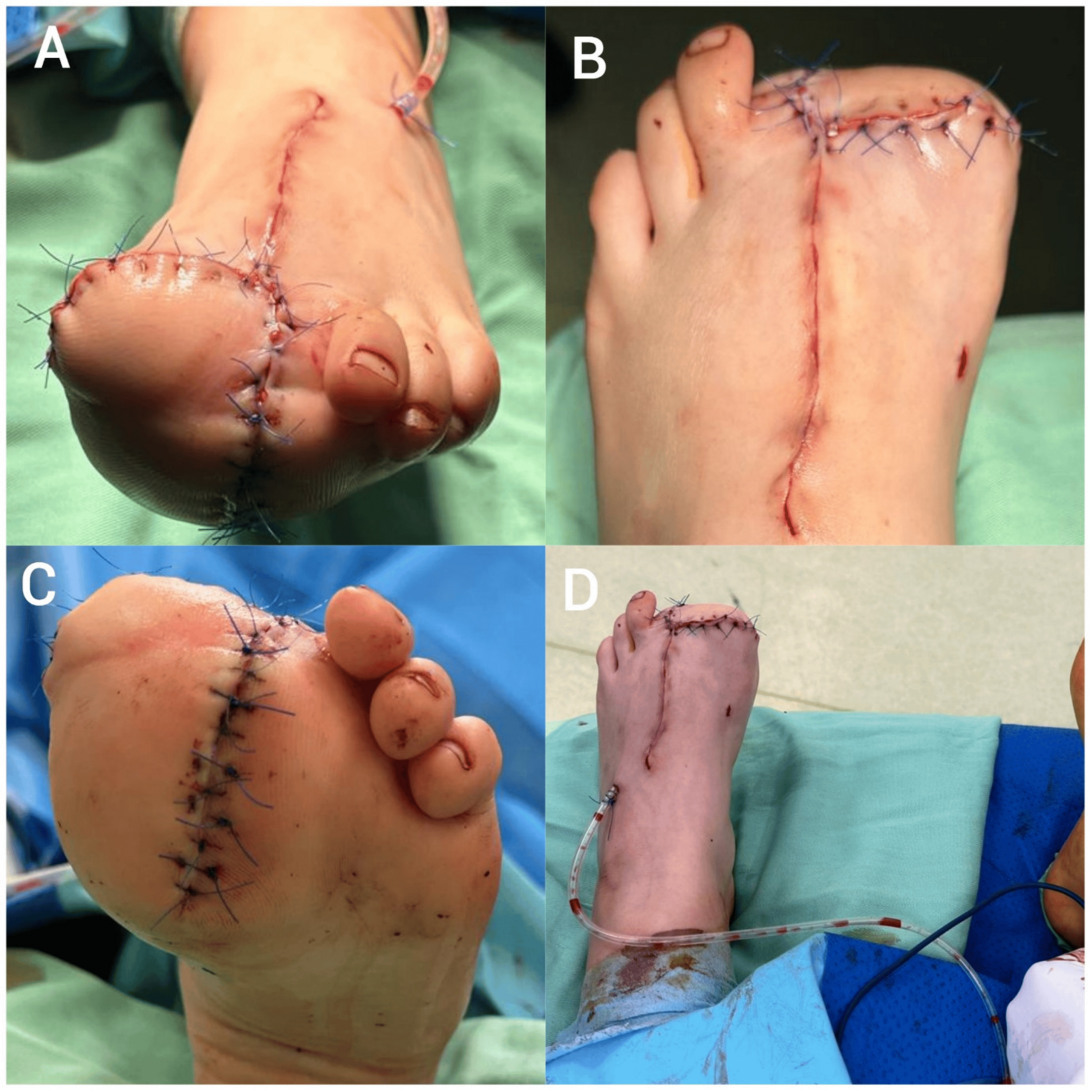
Postoperative clinical appearance. A–anterior view; B–superior view; C–inferior view; D–superior view including post-operative drainage.

The next surgical procedure involved the amputation of the hallux from its proximal third, to perform a reduction of the plantar fat pad and resect the second metatarsal ray. The incision was performed following a triangular shape on the plantar and dorsal surfaces, targeting the second ray from the cuneiform-metatarsal joint. The surgical team tried to remove as much soft tissue as possible, while maintaining the vasculo-nervous bundles. No hypertrophic vasculo-nervous formations or musculature were found upon exploration. The macroscopic intraoperative appearance resembled the compartmentalized fat tissue observed in adults. A fibrous case-like membrane surrounded a large adipose lobule, in which septa divided the lobule into smaller fat areas. The absence of a multitude of small arterioles was noted. Bleeding after using the pneumatic tourniquet was reduced compared to adjacent normal tissue. By extending the incision of the second ray, amputation of the hallux was performed from the proximal third. The base of the proximal phalanx was retained, and the flexor and extensor tendons sutured together. Noteworthy was the thickening of the periosteum of the amputated phalanx and metatarsus. Almost the entire fat pad under the first ray was removed without compromising the blood supply. The first and third metatarsals were approximated together with a non-absorbable suture (Figure 6).

**Figure 7:**
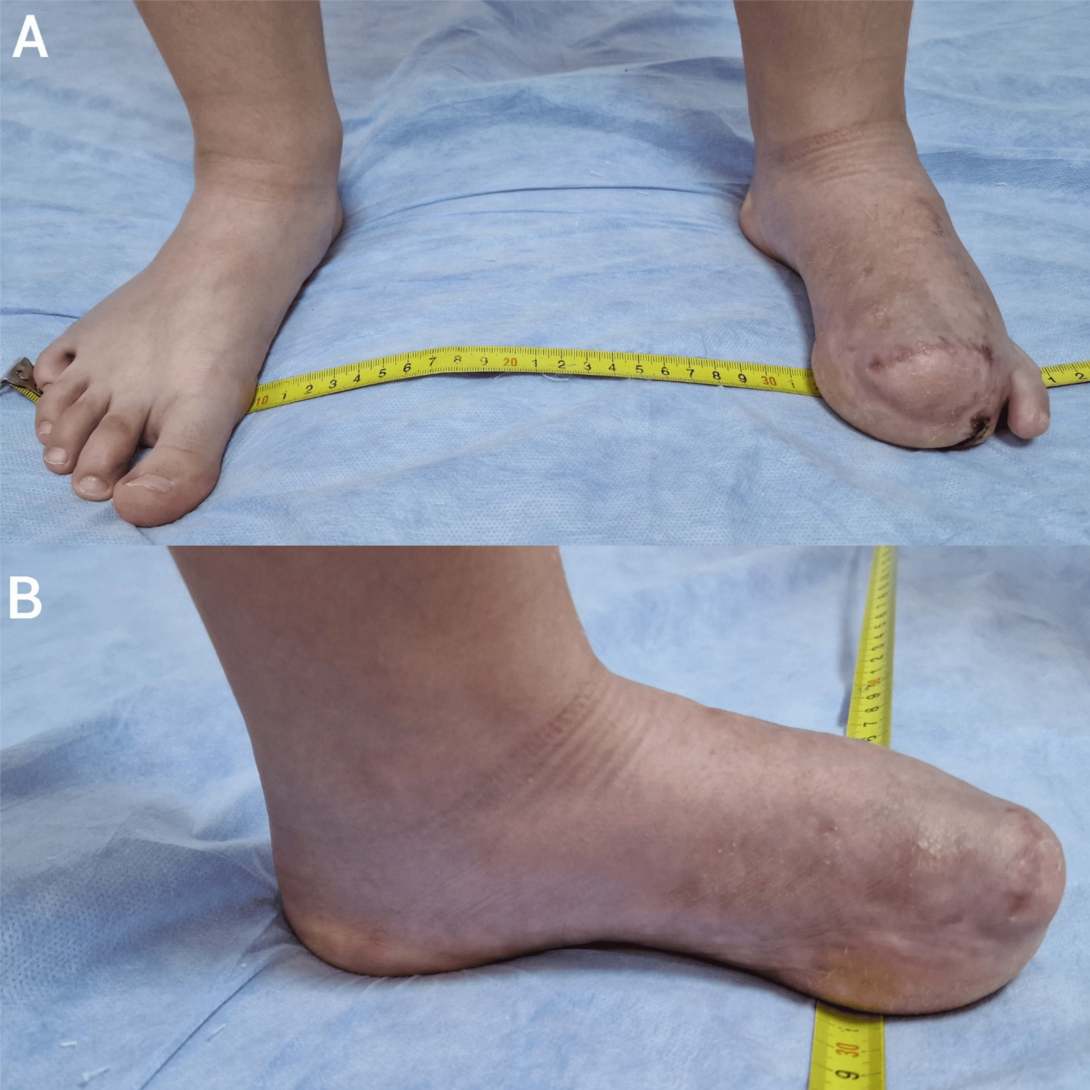
Postoperative clinical appearance at three months. Adequate healing of the amputation site, with a close-to-normal plantar arch and thick medial plantar pad. Left and right foot widths at the level of the metatarsal head are the same. A–anterior view; B–medial view.

A three-week period of rehabilitation followed the operation, ensuring a stress-free course. A mild postoperative complication encountered was the prolonged wound healing time. At three-month postoperative follow-up, the plantar arch and the stepping phase were close to normal, and wearing shoes were unobstructed. The medial plantar pad, still being thicker than normal, functioned as an adequate support surface. The width of the foot at the level of the metatarsal head was the same as on the opposite side (Figure 7).

## Discussion

Probably, the rare character of macrodactyly is the reason why consensus on its epidemiology, clinical presentation, and treatment could not be achieved within the existing body of literature. The majority of these works are either case reports or studies based on a small number of patients. Only a few studies included larger number of patients; namely Wu et al. (170 patients), Chen et al. (93 patients), Wu et al. (73), Barsky (64 patients), Hardwicke et al. (41 patients), Ishida and Ikuta (23 patients), Kotwal and Farooque (23 patients), Chen et al. (26 patients), Kim et al. (18 patients), Wang (12 patients), Krengel et al. (8 patients), Chang et al. (15 patients), Kalen (10 patients), and Dennyson et al. (7 patients) [1,4,5,7,8,10,16,21-27]. There is no prevalence related to gender distribution of toe macrodactyly, even though some authors, namely Kotwal and Farooque, Wu et al. and Chen et al., found this deformity to be more frequent in female patients [8,21,23]. In the study conducted by Hardwicke et al., however, the prevalence was equal among the genders [4].

In more than half of the cases, the left foot is affected, with overgrowth of several toes. The most commonly affected toe is the second one, followed by the third, and then the first. The fourth and fifth toes are only affected in a small percentage. Macrodactyly is usually encountered in areas supplied by a specific nerve; on the foot, this is represented by the innervation area of the medial plantar nerve. Reasons for patients to present to practitioners include difficulties in wearing footwear and finding the appropriate shoes, as well as walking and movement challenges. In the case presented here, the impossibility of finding fitting footwear was further due to the increased dimension of the forefoot, although this was not accompanied by pain or restriction in the patient’s movement or gait. The static form of macrodactyly is reported to be more frequent than the progressive one, as investigated by Chean et al. (63%) and Pearn et al. (87.5%) in their case series [8,28]. If there is absence of comorbidities, the diagnosis is based on clinical presentation and imaging results. Clinical presentation is widely variable. Our patient presented, at the age of three, with the left second toe and hallux being 2.7 cm and 1 cm longer, respectively, as opposed to the corresponding toes of the right foot. The clinical and radiological appearance of the toes and forefoot were roughly proportional, except for the first metatarsus, which was normal in size but presented hypertrophic adipose tissue on the inferior part. In static macrodactyly, since growth remains harmonious, the age of the bone to be corrected should be considered when formulating a surgical plan. The radiographs of our patient showed a smaller opening of growth cartilages in the macrodactyly phalanges and metatarsus. A comparison of the radiographic findings demonstrated that, after the epiphysiodesis, growth on the metatarsus slowed down, but was still greater than on the unaffected bone. A hallux ray ratio of 1.2 was measured at three years of age, and of 1.1 at 10 years of age. Accordingly, the second ray metatarsus ratio decreased from 1.32 to 1.25. Macrodactyly epiphysiodesis bone growth measurements were performed by Kwon et al. Their patient was a six-year-old girl presenting macrodactyly of the second toe of the left foot, in which the left and right second metatarsal lengths were the same (52.8 mm), and the left and right proximal phalanges measured 19.6 mm and 17.5 mm, respectively. The left middle phalanx measured 9.3 mm and the right one 8.0 mm, while the left distal phalanx measured 8.6 mm compared to 7.1 mm of the right one. Epiphysiodesis of the base of the middle phalanx was still considered successful seven years after surgery [29]. For extremely deformed legs, surgical planning is aided by three-dimensional reconstruction from computer tomography, magnetic resonance imaging, and angiographic and lymphographic examinations [5,30]. Its highly variable clinical picture and unpredictable evolution make the management of macrodactyly very demanding. Indeed, setting up a surgical plan aimed to restore functionality and appearance, as close as possible to the regular anatomy, represents a challenge.

In the majority of cases, several surgical techniques are combined and performed in one session. The operations are aimed at reducing soft tissue and bony overgrowth, with the most utilized procedures being excision of overgrown tissue, nail bed excision, ingrown nail plasty, nerve stripping, circumferential bone resection, wedge osteotomy or shortening, toe transplantation, epiphysiodesis, arthrodesis, and amputations. In progressive macrodactyly, multiple surgeries during childhood are to be expected. In fact, a patient who is not radically and early treated will undergo 2.5 to 3.3 surgeries before the growth is complete [4,5,31]. It must be considered that, even after bone growth has ceased, fibroadipose tissue growth may persist; thus, the typical appearance of the toe and foot cannot always be restored, and often, multiple surgical procedures do not produce the desired results. Some authors have hypothesized that it would be advisable to wait until fibroadipose tissue growth is complete, which would lead to a permanent correction [24,32]. Others have suggested that correction in young children represents the best approach, aiming for normal gait development and avoidance of the negative social aspects of the disease [33]. Furthermore, early radical correction, before the onset of walking, is supposed to prevent children from undergoing multiple surgeries and hospital admissions [14].

The average age of the first surgery is around 3.5 years for pediatric macrodactyly patients. Possibly, management of hypertrophic fibroadipose tissue represents the greatest surgical challenge in toe macrodactyly: to exercise as much fibroadipose tissue as possible without compromising the blood supply. For excision of hypertrophic fibroadipose tissue in the forefoot, a longitudinal plantar incision provides good accessibility. Depending on the case, this can be combined with excision of a triangular or elliptical flap. On the toes, a variety and often several incisions are required, according to the deformity. The most used is the dorsal elliptical incision in the midline, which allows good removal of excess tissue, skin, and subcutis. Some surgeons perform a lateral incision for soft tissue and bone reduction in two separate sessions [4]. Circular incisions of the toes, with preservation of neurovascular structures, may also be used to excise varying amounts of skin and subcutis. Sulimann further elaborated this procedure performing two V-shaped vertical incisions in the midline of the plantar and dorsal surfaces. With these, removal of the excess tissue and resection of the middle phalanx segment was carried out. [34]. These results show that reduction of hypertrophic fibroadipose tissue alone represents a solution limited to static macrodactyly. In the progressive form, if the reconstruction treatment is used, further intervention may be required during childhood due to the high recurrence of fibroadipose accumulation [23,35-37].

In their study, Kotwal and Farooque implemented a new technique for 23 patients. Their treatment plan consisted of two stages: the first one was the removal of fibroadipose tissue on the convex side, followed by the same procedure, after three months, on the concave side, combined with the complete removal of the middle phalanx. After the phalangectomy, the distal and proximal interphalangeal joint capsules were sutured, and the toe was stabilized with a Kirschner wire for four to six weeks. Their postoperative complications included axial distortion of the toe due to scar contraction, flap necrosis and, in two cases, subsequent amputation due to aesthetically unpleasant appearance. Lagoutaris et al. performed a two-thirds ostectomy for diaphysis of the proximal phalanx in a three-year-old patient. The ostectomy was stabilized with Kirschner wire for three weeks. They reported good results after 15 years of follow-up [38]. Size reduction and nail preservation are achieved by removing the base of the distal phalanx and the head of the middle phalanx, with subsequent arthrodesis, as developed by Tsuge [39,40]. It is also aimed at preserving the appearance of the toe by flap transplantation of the nail during resection, or its transplantation when distal phalanx amputation is performed [18,41]. Because of subsequent healing difficulties, these techniques should only be used in selected cases. In small children with extremely large deformities, amputations are the treatment of choice, because progressive evolution will lead to eventual loss of correction and, as the digit continues to grow, gigantism is re-established. The level of amputation is determined by the degree of deformity. At the metatarsus, partial or total resection of the corresponding ray, in combination with soft tissue resection, appears to be a valid solution to reduce the macrodactyly size [7,24,33,36,42,43].

The hypertrophic fibroadipose tissue can also be easily removed during ray resection. Several authors have recommended ray resection based on different bone sizes. Natarajan et al. advocates for ray resection for metatarsal opening angles greater than 10 degrees, below which, shortening of the metatarsal diaphysis and soft tissue resection are advisable [44]. Chang et al. recommended ray resection for intermetatarsal values above 10 degrees [24]. In their study, Kim et al. performed ray resection of 18 cases with indications of metatarsal involvement, multiple toe involvement, or stiff toe. The corresponding ray was removed from dorsal and plantar incisions along with the soft tissues. If multiple toes and metatarsus were associated with macrodactyly, ray resection with toe shortening and soft tissue removal was performed. In cases involving three contiguous rays, the middle ray was amputated. A patient in this series presented with postaxial polydactyly associated with macrodactyly of the second and fourth rays; the treatment used here was resection of the third and fourth rays with retention of the extra finger. Their results were deemed satisfactory: 14 patients were satisfied, most of the children could wear the same size of shoes, and 10 patients had mild pain persisting at the level of the surgical scars [25]. Several authors recommend amputation of the corresponding ray as first surgical treatment [33,36]. In their study, Bulut et al. considered ray resection as a single-session definitive solution to avoid subsequent surgery for giant and recurrent macrodactyly [15]. Dennyson et al. also suggested that ray resection was the solution for cosmetically unacceptable cases [7]. Syed et al. described three cases in which resection of the soft tissue and subsequent growth following the first surgery resulted in amputation to improve the foot appearance [45]. In the case of hallux macrodactyly, ray resection is not advisable, the reason being its role while walking. Therefore, phalanx shortening and epiphysiodesis combined with soft tissue resection is recommended [43].

Epiphysiodesis of the macrodactyly bones is also aimed at preservation. When to perform epiphysiodesis is always uncertain due to the age-related advancement of the growth cartilage of the macrodactyly toes compared to normal ones. The simplified rule states that intervention can be carried out when the macrodactyly bone reaches the same size as the corresponding bone of the same-sex parent. Epiphysiodesis technique presents several types, such as removal of the growth cartilage with a Volkmann spoon or osteotomy, followed by stabilization with staples, screws or Kirschner wire. To avoid damage of the neurovascular structures, complete epiphysiodesis is achieved by two operations. Only one side is closed at a time, either plantar or dorsal, and the epiphysiodesis of the other half is performed after three to six months. With the osteotomy technique, bone correction can be executed at the same time. The most common complication of epiphysiodesis is inadequate or unilateral growth cartilage closure with bone axis misalignment. Moreover, complications include an inadequate postoperative toe appearance, but also over-shortening of the metatarsus causing metatarsalgia [46,47]. Macroscopic image of intraoperative tissue shows great variability. Persistent hypertrophy is seen in fibroadipose tissue and bone. Enlargement of other tissues occurs with variable frequency. Chen et al. describe hypertrophy of the intrinsic muscles of the sole in their presentation [8]. In addition, increased bone marrow fat content and excessive thickening of the periosteum have been described [2,33,48].

In the case presented here, normal-sized vasculo-nervous formations were found in the enlarged subcutis. The amputated phalanges and metatarsal periosteum showed thickening. The postoperative result must be judged based on the appearance and function expected by the family and the patient. Ishida and Ikuta tried to quantify the clinical appearance of the foot related to the anatomical one, classifying the mobility and the circumference of the digits [22]. Although in most cases the postoperative appearance of the toe was acceptable, it lost its mobility or became stiff. This stiffness will increase in time. The treatment is considered successful if the patient and the family are satisfied with the appearance. Aesthetically unacceptable toes and forefoot will require another operation. The most common postoperative complications described are prolonged wound healing and necrosis, followed by infection, toe stiffness, shaft misalignment, and contraction of surgical scars, especially after amputation of the second toe [43]. In our patient, the incision showed delayed healing after ray resection; healing was achieved after four weeks.

## Conclusions

Rareness and complexity of clinical presentations of macrodactyly require a tailored treatment plan which usually involves several surgical procedures to achieve both functional and cosmetic outcomes. The progression of this condition makes therapy and resolution more challenging, particularly in cases when growth irregularities aggravate the deformity over time.

Effective management of macrodactyly depends on a patient-centered approach considering the unique anatomical and functional needs of every individual. While surgical treatments could result in significant improvements, they usually compromise mobility and appearance. Treatment planning should thus involve a thorough discussion with the patient and their family about pragmatic expectations and probable results. Given the importance of optimizing treatment plans and enhancing patient outcomes, further research and clinical experience are essential to refining management strategies for macrodactyly.
